# A new fluorescence-based optical imaging method to non-invasively monitor hepatic myofibroblasts *in vivo*

**DOI:** 10.1016/j.jhep.2016.03.021

**Published:** 2016-07

**Authors:** Saimir Luli, Daniela Di Paolo, Patrizia Perri, Chiara Brignole, Stephen J. Hill, Helen Brown, Jack Leslie, H.L. Marshall, Matthew C. Wright, Derek A. Mann, Mirco Ponzoni, Fiona Oakley

**Affiliations:** 1 Fibrosis Research Group, Institute of Cellular Medicine, Newcastle University, Newcastle upon Tyne NE2 4HH, UK; 2 Experimental Therapy Unit, Laboratory of Oncology, Istituto Giannina Gaslini, Genoa, Italy

**Keywords:** HM, hepatic myofibroblast, αSMA, alpha-smooth muscle actin, WT, wild-type, ScAb, single chain antibody, CCl_4_, chronic carbon tetrachloride, NIR, near infra-red, ROI, region of interest, ALT, alanine transaminase, AST, aspartate aminotransferase, PBS, phosphate buffered saline, BDL, bile duct ligation, ALP, alkaline phosphatase, DOX, Doxorubin, SYN, synaptophysin, Non-invasive imaging, Hepatic myofibroblasts, Fibrosis, Cell-targeting, Mouse

## Abstract

**Background & Aims:**

Currently, staging of fibrosis in preclinical rodent liver fibrosis models is achieved histologically. Many animals are used at multiple time-points to assess disease progression or therapeutic responses. Hepatic myofibroblasts promote liver fibrosis therefore quantifying these cells *in vivo* could assess disease or predict therapeutic responses in mice. We fluorescently labelled a single chain antibody (C1-3) that binds hepatic myofibroblasts to monitor fibrogenesis *in vivo*.

**Methods:**

CCl_4_ was used to induce acute liver injury in WT and *cRel*^−/−^ mice. Bile duct ligation was used to model chronic fibrosis. Hepatic myofibroblasts were depleted using a liposome-drug delivery system or chemically with sulfasalazine. An IVIS® spectrum visualised fluorophore-conjugated C1-3 *in vivo*.

**Results:**

IVIS detection of fluorescently labelled-C1-3 but not a control antibody discriminates between fibrotic and non-fibrotic liver in acute and chronic liver fibrosis models. *cRel*^−/−^ mice have a fibro-protective phenotype and IVIS signal is reduced in CCl_4_ injured *cRel*^−/−^ mice compared to wild-type. *In vivo* imaging of fluorescently labelled-C1-3 successfully predicts reductions in hepatic myofibroblast numbers in fibrotic liver disease in response to therapy.

**Conclusions:**

We report a novel fluorescence imaging method to assess murine hepatic myofibroblast numbers *in vivo* during liver fibrosis and after therapy. We also describe a novel liposomal antibody targeting system to selectively deliver drugs to hepatic myofibroblasts *in vivo*. C1-3 binds human hepatic myofibroblast therefore imaging labelled-C1-3 could be used for clinical studies in man to help stage fibrosis, demonstrate efficacy of drugs that promote hepatic myofibroblast clearance or predict early therapeutic responses.

**Lay summary:**

In response to damage and injury scars develop in the liver and the main cell that makes the scar tissue is the hepatic myofibroblast (HM). C1-3 is an antibody fragment that binds to the scar forming HM. We have fluorescently labelled C1-3 and given it to mice that have either normal or scarred livers (which contain HM) and then used a machine called an *in vivo* imaging system (IVIS) that takes pictures of different wavelengths of light, to visualise the antibody binding to HM inside the living mouse. Using fluorescently labelled C1-3 we can assess HM numbers in the injured liver and monitor response to therapy. We have also used C1-3 to target drugs encapsulated in lipid carriers (liposomes) to the HM to kill the HM and reduce the liver disease.

## Graphical abstract

**Figure:**
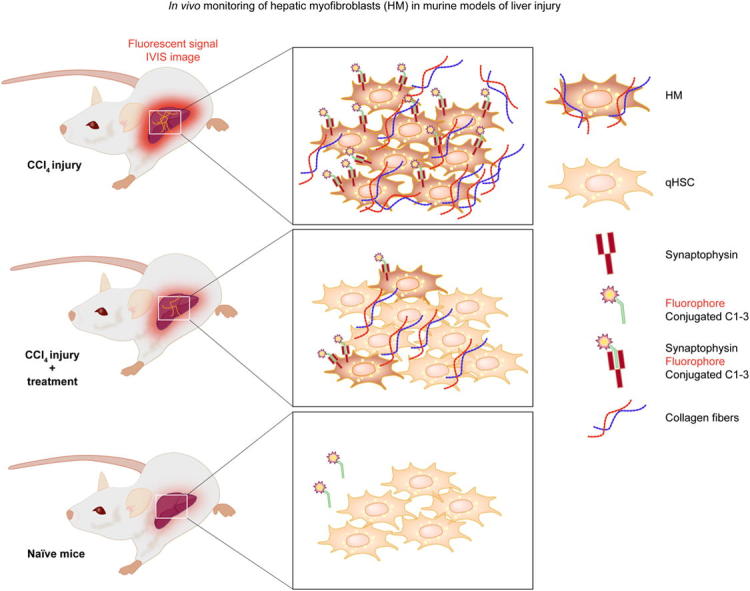

## Introduction

Liver fibrosis is characterised by excess deposition of collagens by the primary scar-producing cell in the liver the hepatic myofibroblast (HM) [1]. HM are generated from quiescent hepatic stellate cells (qHSC) upon injury via a process called activation [2]. In acute liver injury HM produce a temporary scar to allow wound healing and once the liver has regenerated and normal homeostasis is restored, the HM are cleared by apoptosis or undergo de-differentiation [3]. In chronic injury HM persist, migrate and proliferate promoting scar formation and fibrosis [4], [5]. Liver fibrosis is a highly dynamic process that can either progress or resolve. The HM is a key cell type regulating the kinetics of fibrosis and fibrolysis. HM accumulate during progression of liver fibrosis but their clearance precedes fibrolysis and remodeling of the scar matrix [6], [7], [8]. If a drug successfully treats the underlying cause of injury, promotes HM apoptosis or if the injury stimulus is removed the scar is remodeled [6], [9].

Currently, histologically assessing fibrosis (Sirius Red) and HM numbers (alpha-smooth muscle actin (αSMA)) in the liver is the only accurate method to stage fibrosis in murine preclinical liver fibrosis [5], [10], [11]. Therefore large numbers of animals are used for multiple time-points to monitor disease kinetics or test novel anti-fibrotic drugs. Developing a method to image and assess HM *in vivo* would allow researchers to perform minimally invasive longitudinal monitoring of fibrosis progression or resolution. This could reduce the number of mice required to perform liver disease models when comparing wild-type (WT) and transgenic mice or predict early therapeutic responses when testing anti-fibrotic drugs.

Synaptophysin mRNA is detected in rat quiescent hepatic stellate cells (qHSC) and cultured HM. Immuno-histochemical staining demonstrated co-localisation of synaptophysin and αSMA in fibrotic liver [12]. C1-3-GT has previously been used to deplete HM from the livers of acute and chronic carbon tetrachloride (CCl_4_) injured mice [13], proving that C1-3 binds and successfully delivers drugs to HM *in vivo*. However, this drug targeting approach is limited by suitability of the drug in conjugation chemistry. This study aims to exploit the HM binding properties of C1-3 to develop two novel tools for fibrosis research. Firstly, develop a novel imaging probe to monitor HM *in vivo*. Secondly, conjugate C1-3 to liposomal carriers to selectively deliver drugs to HM *in vivo*.

## Materials and methods

### C1-3 Production and purification

pIMS-147-C1-3 plasmid transformed E-Coli XL-1 blue cells were cultured in Lysogeny broth media containing antibiotics (ampicillin and tetracycline). E-Coli expressing pIMS-147-C1-3 were cultured in Luria-Bertani broth overnight then in Terrific Broth media for 8 h. C1-3 expression was induced by adding isopropyl β-D-1-thiogalactopyranoside to culture media for 4 h. Cells were lysed and C1-3 was purified from the supernatant using immobilised metal affinity chromatography as previously described [13], [14], [15]. Endotoxin was removed using Q maxi H columns (Sartorious Vivascience).

### Liposomal preparation

C1-3 or CSBD9 coated sterically stabilised liposomes loaded with doxorubicin were prepared as previously described [16]. Full details in Supplementary materials and methods.

### Mice and models of liver injury

All experiments were performed on male C57BL/6 (WT) or global *cRel* knockout mice (*cRel*^−/−^) mice under approval from the Newcastle Ethical Review Committee and a UK Home Office licence. Acute liver damage was induced by intraperitoneal injection of a single of dose of CCl_4_ at 2 μl/g body weight (CCl_4_:olive oil at 1:1 [vol/vol]). Bile duct ligation (BDL) was achieved by surgically exposing the common bile duct followed by its double ligation. Appropriate pain relief was given to all mice. Mice developed chronic liver fibrosis for a period of 14 days [17].

### Therapies

At 24 h post CCl_4_ injury animals were intravenously injected with C1-3 or CSBD9 coated sterically stabilised liposomes loaded with 5 mg/kg doxorubicin or empty liposomes. Free doxorubicin was given at a concentration of 5 mg/kg. Chemical depletion of HM was achieved by intraperitoneal injection of a single of dose sulfasalazine (150 mg/kg) 24 h post injury.

### *In vivo* fluorescent imaging

Conjugation of C1-3 or CSBD9 to DyLight800 (Thermo Scientific) fluorophore was performed following manufactures instruction. Sufficient quantities of C1-3 was produced and labelled prior to each individual experiment to control for antibody batch-to-batch variation or differences in labelling efficiency. Mice under isoflurane were fluorescently imaged (745/800 em/ex filters) using epi-fluorescence on an IVIS spectrum (Caliper Life Sciences) at 2 h, 4 h and 6 h post *i.v.* injection of Dylight800 labelled-C1-3 for biodistribution studies. For all other experiments the *in vivo* imaging of fluorescent labelled-C1-3 or CSBD9 was performed at 6 h post C1-3/CSBD9 (10 mg/kg) administration. After the final scan mice were humanely killed and the liver, kidney and spleen were excised and IVIS imaged (745/800 em/ex filters). Images were analysed using Living Image 4.3.1 software, regions of interest (ROI) were drawn as described in the Supplementary methods section and average radiant efficiency [p/s/cm^2^/sr]/[μW/cm^2^] was calculated after subtracting the background signal.

### Statistical analysis

Data was analysed using Excel or GraphPad Prism, *p* values were calculated using a two-tailed unpaired Student *t* test or one-way ANOVA with Newman-Kuels post hoc test and *p* <0.05 (∗), *p* <0.01 (∗∗) or *p* <0.001 (∗∗∗) was considered significant.

## Results

We show that synaptophysin expression is increased in culture-activated and *in vivo* activated mouse HM compared to qHSC (Supplementary Fig. 1A–C). The single chain antibody (ScAb) C1-3 recognises an extracellular domain in synaptophysin, a protein expressed on HM in the liver. C1-3 has been reported to bind to HM but not qHSC or other liver cell types [12], [14], [15]. We show binding of C1-3 to mouse and human HM in culture (Supplementary Fig. 1D) and report that the pro-apoptotic molecule gliotoxin (GT) induces death (reduced cell attachment) of both quiescent HSC and HM (Supplementary Fig. 1E–F). However, when GT is conjugated to C1-3 (C1-3-GT) this complex is ∼25 fold less potent at inducing death of qHSC (C) than HM (D), suggesting that C1-3 efficiently targets and kills HM.

CCl_4_ metabolism by the liver produces hepatotoxic free radicals [14]. Administration of one dose of CCl_4_ to mice promotes hepatocyte death and induces an inflammatory response, which drives HM activation [18], [19]. HM numbers peak between 48-72 h with the majority of HM then cleared from the liver by either apoptosis or de-differentiation by day 5 [20], [21], [22].

We wanted to establish the biodistribution and clearance kinetics of C1-3 labelled with a near infra-red (NIR) fluorescent dye (DyLight800) in mice with acute liver injury. 48 h acute CCl_4_ injured WT C57/Bl6 mice were administered NIR fluorophore-conjugated-C1-3 via the tail-vein followed by whole body IVIS imaging at 2, 4, 6, 12 h, and 1 day. *In vivo* fluorescence signal peaks at 2 h and then rapidly falls between 2-6 h. A further decrease occurs between 6-12 h with the signal plateauing between 12-24 h (Supplementary Fig. 2A). *Ex vivo* imaging of organs at 48 h post CCl_4_ revealed that the fluorescence signal was highest in the liver and little or no signal was detected in the heart, spleen, pancreas or brain (Supplementary Fig. 2B). ScAb are cleared by the kidneys and excreted into the urine [14], [15] therefore we expect to detect some fluorescence signal in the *ex vivo* imaged kidney (Supplementary Fig. 2C). From the biodistribution experiments we decided to image mice at 6 h for future studies for three reasons: i) The majority of unbound antibody has been excreted; ii) there is a strong signal from the liver; iii) this time-point has the least mouse-to-mouse variation in fluorescence signal (Supplementary Fig. 2A).

**Fig. 1:**
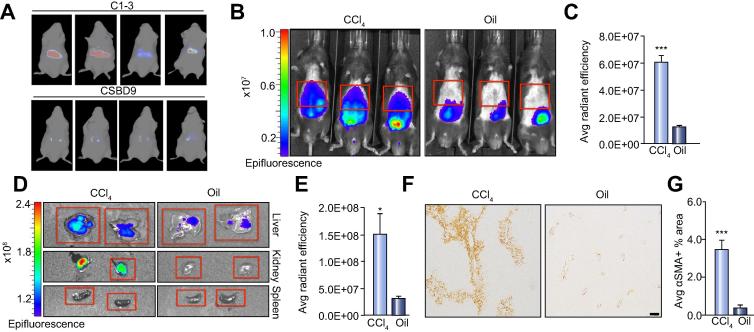
**Optical imaging of fluorescently labelled-C1-3 non-invasively monitors hepatic myofibroblasts in mice during acute liver injury.** (A) Representative 2D IVIS pictures of Fluorescent Imaging Tomography (FLIT) imaging of C1-3 or CSBD9 in 48 h CCl_4_ injured mice. (B) Whole body IVIS images of acute CCl_4_ and olive oil (oil) injured mice. Red boxes show ROI. (C) Graph showing average radiant efficiency [p/s/cm^2^/sr]/[μW/cm^2^] of IVIS imaged mice. (D) E*x vivo* images of liver, kidney and spleen. (E) Graph showing average radiant efficiency. (F) Photomicrographs (3 × 3 fields, 100× magnification) of αSMA stained liver sections (scale bar, 200 μ). (G) Graph showing average αSMA+ area in CCl_4_ and oil injured mice. Data are means ± s.e.m, minimum n = 3/group. Unpaired *t* test, ∗*p* <0.05 or ∗∗∗*p* <0.001 compared to oil control.

To verify that IVIS signal from fluorescently labelled C1-3 originates from the liver after injury we performed 2D fluorescence imaging (Supplementary Fig. 3A–B) and Fluorescent Imaging Tomography (FLIT) imaging (Fig. 1A), which uses geometry, depth and intensity of the signal to build a 3D reconstruction of the mouse and provide an anatomical localisation of the fluorescence signal. 3D FLIT images show retention of fluorescently labelled-C13 in the liver (Supplementary Movie 1), conversely, signal from fluorescently labelled-CSBD9, a control ScAb predominantly originates from the kidney with a low level of fluorescence detected in the liver (Supplementary Movie 2). CSBD9 has an identical antibody backbone to C1-3 but recognises a bacterial protein and therefore should not bind mammalian proteins. Analysis of αSMA staining confirmed that the livers of C1-3 and CSBD9 imaged mice contained similar numbers of HM (Supplementary Fig. 3C).

To determine if IVIS imaging of fluorescently labelled-C1-3 could be used to monitor HM *in vivo*, we administrated labelled-C1-3 intravenously to olive oil, vehicle treated mice or acute CCl_4_ injured WT mice and then performed IVIS imaging. Fluorescently labelled-C1-3 discriminates between normal (olive oil) and acute CCl_4_ injured mice where HM are activated. Representative IVIS images show a higher fluorescent signal from the upper abdominal area where the liver is anatomically located in acute CCl_4_ injured mice compared to olive oil control mice (Fig. 1B). The average radiant efficiency was quantified by drawing a ROI, denoted by red boxes (Fig. 1B), over the upper abdomen. Fig. 1C shows a highly significant increase in fluorescence intensity in the CCl_4_ injured mice compared to olive oil mice. To verify that the fluorescence signal originates from the liver we removed the liver, kidney and spleen and performed *ex vivo* imaging. The representative IVIS images and graph show that the fluorescence intensity is significantly higher in livers of CCl_4_ injured mice than olive oil mice, with the signal being completely lost in the olive oil group at 6 h post C1-3 administration (Fig. 1D–E). As expected the histological levels of αSMA is significantly increased in the CCl_4_ injured mice compared to olive oil mice (Fig. 1f–g). Liver injury in acute CCl_4_ treated mice was confirmed by elevation of the serum transaminases alanine transaminase (ALT) and aspartate aminotransferase (AST) compared to the olive oil treated mice (Supplementary Table 1).

To exclude the possibility that there is non-specific retention or non-specific hepatic clearance of the fluorescently labelled-C1-3 in the injured liver compared to the olive oil uninjured liver we directly compared imaging of C1-3 with CSBD9 (control ScAb). 48 h CCl_4_ injured WT mice were given either fluorescently labelled-C1-3 or CSBD9 and then IVIS imaged. We observe strong fluorescence signal from the upper abdominal area of mice given fluorescently labelled-C1-3 but no or low signal in the fluorescently labelled-CSBD9 group (Supplementary Fig. 4A). The difference in fluorescence signal is highly significant when quantified as average radiant efficiency (Supplementary Fig. 4B). *Ex vivo* epifluorescence imaging of the liver, kidney and spleen confirmed that the fluorescence signal originated from the liver (Supplementary Fig. 4C). A high liver signal is detected in the C1-3 group and no signal is detected in the CSBD9 treated animals, ruling out non-specific binding of the ScAb (Supplementary Fig. 4D). Serum transaminases ALT and AST were elevated equally in both groups of mice and the αSMA+ stained area in liver sections from the C1-3 and CSBD9 imaged animals was similar, confirming that the liver injury and HM activation was comparable in both groups (Supplementary Table 1 and Supplementary Fig. 4E–F).

**Fig. 2:**
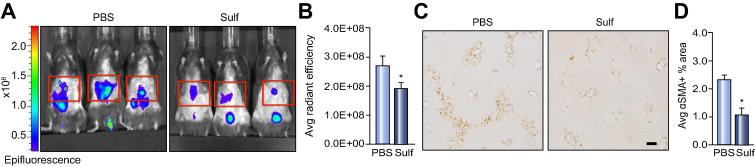
**Chemical depletion of hepatic myofibroblasts can be monitored in acute liver injury by C1-3.** (A) Whole body IVIS images of acute CCl_4_ injured mice treated with PBS vehicle or sulfasalazine (Sulf). Red boxes show ROI used to calculate the average radiant efficiency. (B) Graph showing Average Radiant Efficiency of PBS or sulfasalazine treated acute CCl_4_ injured mice. (C) Photomicrographs at 100× magnification (3 × 3 fields) of αSMA stained liver sections. (D) Graph showing average αSMA+ stained area in PBS or sulfasalazine treated acute CCl_4_ injured mice. (scale bar, 200 μ). Data are means ± s.e.m, minimum n = 6/group. Unpaired *t* test, ∗*p* <0.05 compared to PBS control.

To ascertain if C1-3 imaging detects a therapeutic response in acute liver injury we injured mice with CCl_4_ and then at 24 h, once HM have activated we gave a single intraperitoneal dose of sulfasalazine, a compound previously shown to induce HM apoptosis *in vitro* and *in vivo* or phosphate buffered saline (PBS) Veh control [23]. 24 h later mice were given fluorescently labelled-C1-3 and IVIS imaged. The representative IVIS pictures and average radiant efficiency graph show a statistically significant reduction in epifluorescence in sulfasalazine treated mice compared to the PBS control (Fig. 2A–B). Correlation analysis shows a positive relationship (R^2^ = 0.70) between IVIS signal and αSMA+ area (Supplementary Fig. 4G) between untreated and sulfasalazine treated mice. The degree of liver injury quantified by increased ALT and AST levels was similar in both groups (Supplementary Table 1) but HM numbers determined by hepatic αSMA+ staining from sulfasalazine treated mice was reduced by ∼50% compared to WT (Fig. 2C–D).

**Fig. 3:**
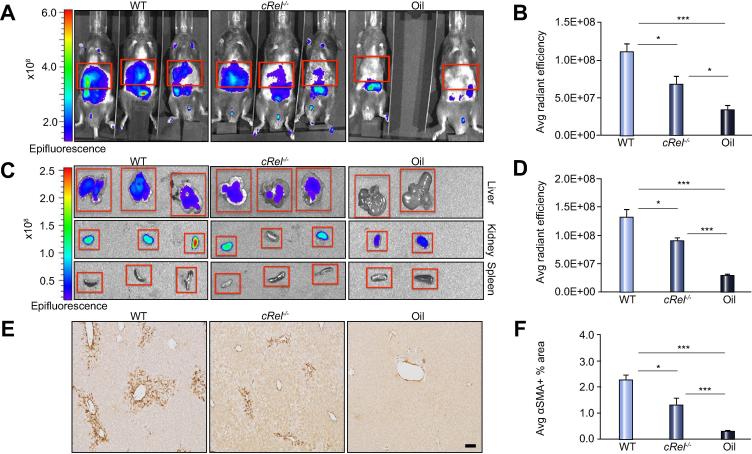
**Optical imaging of fluorescently labelled-C1-3 detects differences in hepatic myofibroblast numbers in wild-type *vs.* transgenic mice after acute liver injury.** (A) Whole body IVIS images and (B) graph showing average radiant efficiency in acute CCl_4_ injured WT and *cRel*^−/−^ mice and oil controls. (C) Images of liver, kidney, and spleen. (D) Graph showing average radiant efficiency of *ex vivo* imaged CCl_4_ and oil livers. (E) Photomicrographs (100× magnification, 3 × 3 fields) of αSMA staining. (F) Graph showing average αSMA+ stained area in acute CCl_4_ injured and oil treated WT and *cRel*^−/−^ mice. Data are means ± s.e.m, minimum n = 8/group, scale bar, 200 μ. ANOVA, ∗*p* <0.05 or ∗∗∗*p* <0.001 compared to WT.

We previously reported that global *cRel* knockout mice (*cRel*^−/−^), which lack the c-Rel subunit of the NF-κB transcription factor family, are protected from developing liver fibrosis and that fewer HM become activated in the liver of *cRel*^−/−^ mice during acute or chronic liver injury [24]. To determine if our novel imaging method is sensitive enough to detect differences in HM activation between WT and transgenic mice we acute CCl_4_ injured WT and *cRel*^−/−^ mice and then performed IVIS imaging. The *in vivo* epifluorescence signal was significantly increased in WT mice compared with *cRel*^−/−^ mice (Fig. 3A–B) despite no difference in liver transaminases (Supplementary Table 1). Correlation analysis revealed a strong relationship (R^2^ = 0.88) between IVIS signal and αSMA+ area (Supplementary Fig. 4H), between olive oil treated WT mice and acute CCl_4_ injured WT and *cRel*^−/−^ mice. *Ex vivo* imaging of the liver, kidney and spleen proved that the fluorescence signal originated from the liver (Fig. 3C–D). As expected there was a significant reduction in αSMA+ HM in the livers of *cRel*^−/−^ mice compared to WT (Fig. 3E–F) confirming that the IVIS signal correlated with HM numbers. When both strains of injured mice were compared to olive oil controls, there was an incremental increase in both *in vivo* and *ex vivo* IVIS signal between olive oil, *cRel*^−/−^ and WT mice as HM increase (Fig. 3A–F).

Taken together these data suggest that C1-3 can be used to non-invasively image HM *in vivo* during acute liver injury, predict a successful therapeutic response of an anti-fibrotic compound and screen transgenic mice for fibro-protective phenotypes. We next wanted to ask; can C1-3 monitor HM accumulation and progression of fibrosis in chronic liver injury?

**Fig. 4:**
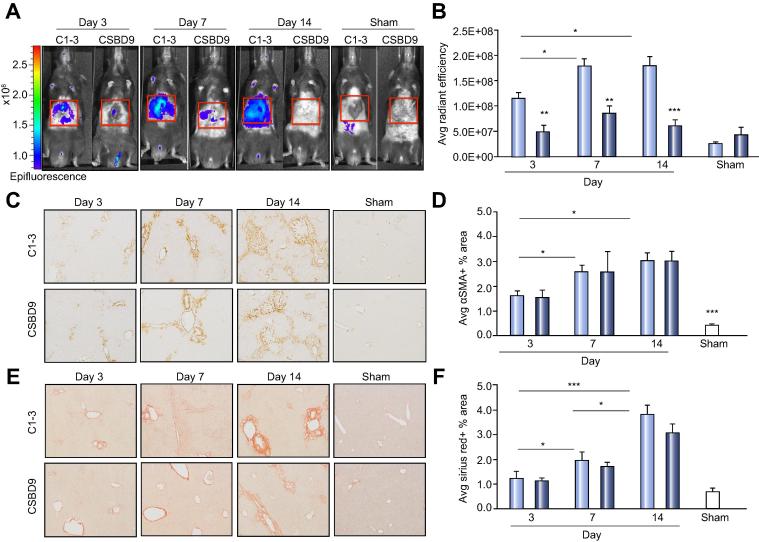
**Optical imaging of fluorescently labelled-C1-3 signal correlates with hepatic myofibroblasts numbers during chronic liver injury.** (A–B) IVIS images and graph showing average radiant efficiency of day 3, 7 and 14 BDL or sham mice, imaged with fluorescently labelled-C1-3 or CSBD9. Imaging data; C1-3-BDL day 3 (n = 5), day 7 (n = 6), day 14 (n = 8) and CSBD9-BDL day 3 (n = 4), day 7 (n = 5), day 14 (n = 8). (C) Photomicrographs of αSMA staining and (D) graph showing average αSMA+ area in BDL or sham mice. (E) Photomicrographs of Sirius Red stained liver sections and (F) graph showing average Sirius Red+ area in BDL or sham mice. Histology data are means ± s.e.m, in C1-3-BDL day 3 (n = 4), day 7 (n = 4), day 14 (n = 8) and CSBD9-BDL day3 (n = 4), day 7 (n = 4), day 14 (n = 8). ANOVA, ∗*p* <0.05, ∗∗*p* <0.01 or ∗∗∗*p* <0.001 *vs.* WT.

BDL is a progressive liver fibrosis model [17], [25]. We performed BDL or a sham operation and then gave fluorescently labelled-C1-3 or CSBD9 and IVIS imaged mice at day 3, day 7 and day 14. IVIS images show a significant increase in epifluorescence signal in C1-3 imaged BDL mice as disease progresses from early (day 3), established (day 7) and advanced (day 14) fibrosis. Conversely no or low signal was detected in the CSBD9 imaged BDL mice or in the sham operated mice given either labelled C1-3 or CSBD9 (Fig. 4A). The low level of fluorescence signal in the CSBD9 group is likely due to slightly altered clearance kinetics in chronic injured mice and/or due to physiological differences between the BDL and acute CCl_4_ models. Serum transaminases, ALT, AST and alkaline phosphatase (ALP) a marker of biliary damage and the number of CK19+ biliary cells were similar between BDL mice imaged with C1-3 and CSBD9 at each time-point suggesting that there was no difference in liver injury or ductular reaction between the two groups as the model progressed (Supplementary Table 1 and Supplementary Fig. 5A). IVIS signal is statistically significantly different between BDL mice imaged with C1-3 and CSBD9 at all time-points or when C1-3 BDL mice are compared to sham operated animals (Fig. 4B). However, there are no differences in fluorescence intensity between sham operated control mice and the CSBD9 imaged BDL mice.

HM numbers and fibrosis severity were histologically assessed using αSMA immuno-histochemistry and Sirius Red staining. αSMA+ area was similar at each time-point between C1-3 and CSBD9 imaged mice, therefore the reduction in fluorescence signal in the CSBD9 imaged BDL group is not due to fewer HM (Fig. 4C-D). HM were present by day 3 and numbers significantly increased at day 7 but plateaued by day 14, interestingly there was no difference in IVIS signal in C1-3 imaged mice from day 7 to day 14, suggesting that the epifluorescence accurately predicted the number of HM in the liver. Indeed, correlative analysis of average radiant efficiency *vs.* αSMA+ area shows positive correlation (R^2^ = 0.79) in fluorescence signal between sham animals and BDL mice (Supplementary Fig. 5B). Fibrotic scar matrix steadily accumulates from day 3 to day 14 and there are no differences in Sirius Red stained area between C1-3 and CSBD9 imaged BDL mice (Fig. 4E–F). Fluorescence signal correlates poorly with collagen (R^2^ = 0.29) providing additional evidence that C1-3 selectively binds HM and does not non-specifically bind to collagen matrix (Supplementary Fig. 5C).

**Fig. 5:**
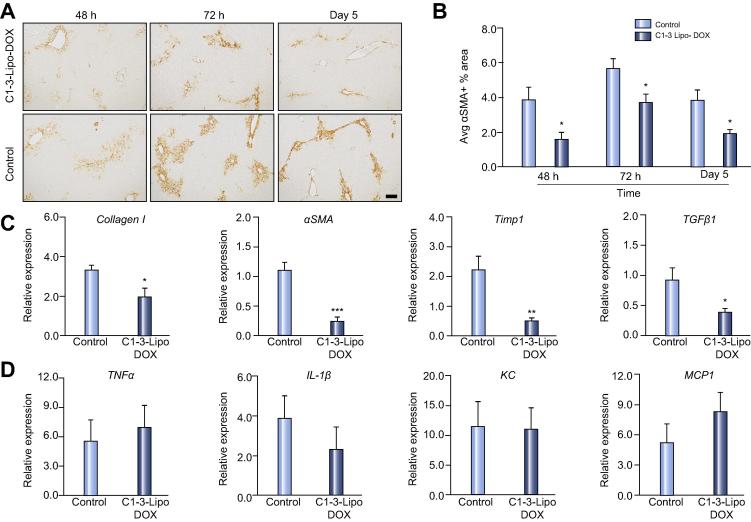
**C1-3-liposomes can selectively deliver doxorubicin to deplete HM during acute liver injury.** (A–B) Photomicrographs and graph showing average αSMA+ stained area in αSMA stained liver sections from 48 h-5 day acute CCl_4_ injured WT mice treated with C1-3 coated liposomes containing doxorubicin (Lipo-DOX) or empty (Control). Relative hepatic mRNA expression of *collagen I*, *αSMA*, *TIMP1* and *TGFβ1* (C) and *TNFα*, *IL-1β*, *KC* and *MCP1* (D) in 72 h acute CCl_4_ injured WT mice treated with either control or DOX loaded C1-3 coated liposomes. Data are means ± s.e.m, minimum of n = 5/group. Unpaired *t* test, ∗*p* <0.05, ∗∗*p* <0.01 or ∗∗∗*p* <0.001 compared to control.

Global *cRel* gene knockout mice or systemic administration of sulfasalazine reduces HM numbers in the injured liver. However, these HM depleting approaches are not selective and may have unexpected affects on IVIS imaging. We therefore wanted to test whether C1-3 imaging can monitor targeted depletion of HM from injured livers of WT mice. Liposomes provide a unique system to encapsulate drugs and when complexed to targeting molecules e.g., an antibody or receptor ligand can selectively deliver therapeutic agents to specific cells or organs *in vivo* [16], [26], [27]. Therefore targeted liposomal drug delivery can improve efficacy and reduce side effects. Doxorubicin therapy reduces fibrosis in BDL injured rats [28]. However, this drug has cytotoxic effects at high concentrations. A single dose of 5 mg/kg free doxorubicin in acute CCl_4_ injury caused a modest but insignificant increase in serum liver enzymes and a small but insignificant reduction in αSMA+ area (Supplementary Fig. 6A–B). To selectively deplete HM *in vivo* during acute liver injury we have exploited the HM binding properties of C1-3 and developed a new method to target liposomes encapsulating 5 mg/kg doxorubicin to HM. C1-3-coated liposomes but not uncoated liposomes (control) bind to mouse and human HM in culture but primary hepatocytes or the Raji B cell line (Supplementary Fig. 6C). Mice were injured with CCl_4_ and then given C1-3-Doxorubin-liposomes (C1-3-Lipo-DOX) or C1-3-empty-liposomes (control) 24 h later. Liver and blood samples were then harvested at 48 h, 72 h and 5 days post CCl_4_. Liver enzymes ALT, AST and ALP were elevated equally in both groups suggesting that DOX liposomes did not exacerbate liver injury (Supplementary Table 1). αSMA+ area was significantly reduced at all time-points in the C1-3-Lipo-DOX treated animals compared to the control liposomes, confirming that this method depletes HM *in vivo* in acute liver injury (Fig. 5A–B). As expected, hepatic expression of the pro-fibrotic genes collagen I, αSMA, TIMP1 and TGFβ1 were significantly decreased at 72 h in mice that received Lipo-DOX (Fig. 5C). Importantly, C1-3-Lipo-DOX did not affect the inflammatory response to acute liver injury with neutrophil (NIMP-R14) and T-lymphocyte (CD3) recruitment and macrophage (F4/80) numbers being similar between both groups (Supplementary Fig. 6D–E). This was consistent with no difference in hepatic expression of the inflammatory genes TNFα, IL-1β, KC and MCP1 in control or DOX liposome treated mice (Fig. 5D). HM have been reported to suppress liver regeneration after injury [29]. Interestingly, at day 5 the DOX treated mice have a significantly higher proliferative rate than mice treated with control liposomes (Supplementary Fig. 6G). These data prove that C1-3-liposomes can be used to deliver apoptosis-inducing drugs to HM and promote their clearance *in vivo*. We next repeated this study and performed IVIS imaging to prove that fluorescently labelled-C1-3 can monitor targeted depletion of HM. 24 h post CCl_4_ injury mice were given vehicle control, empty liposome (Empty-Lipo) control, uncoated liposomes containing DOX (Lipo-DOX) control or C1-3 coated liposomes encapsulating DOX to specifically target and deplete HM (C1-3-Lipo-DOX) followed by IVIS imaging 48 h later. Both *in vivo* and *ex vivo* imaging revealed that the fluorescent C1-3 IVIS signal was only reduced in the C1-3-Lipo-DOX group (Supplementary Fig. 7A–B). HM depletion in mice receiving C1-3-Lipo-DOX was confirmed by a reduction in αSMA+ area (Supplementary Fig. 7C) and there was a positive correlation between αSMA+ area and C1-3 IVIS signal, R^2^ = 0.77 (Supplementary Fig. 7D). Serum transaminases were elevated equally in all groups (Supplementary Table 1).

**Fig. 6:**
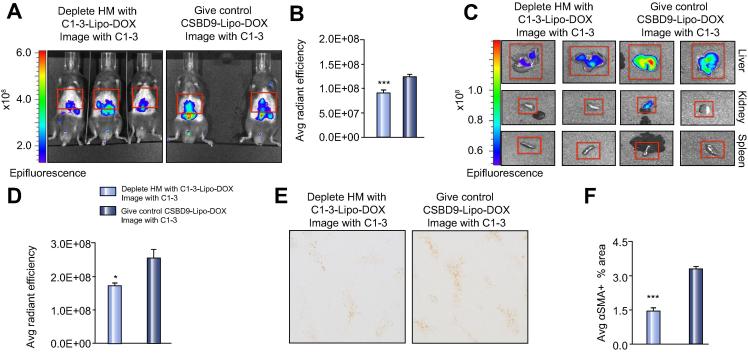
**Targeted depletion of hepatic myofibroblasts in acute liver injury can be monitored using optical imaging of fluorescently labelled-C1-3.** (A–B) IVIS images and graph showing average radiant efficiency in acute CCl_4_ injured WT mice given DOX containing liposomes (C1-3-lipo or CSBD9-lipo). (C) E*x vivo* images of liver, kidney and spleen. (D) Graph (average radiant efficiency) of *ex vivo* imaged livers in C1-3-lipo or CSBD9-lipo treated mice. (E) Photomicrographs (100× magnification, 3 × 3 fields), scale bar, 200 μ. (F) Graph showing average αSMA+ area in acute CCl_4_ injured WT mice given C1-3-lipo or CSBD9-lipo groups. Data are means ± s.e.m, minimum of n = 6/group. Unpaired *t* test, ∗*p* <0.05 or ∗∗∗*p* <0.001 compared to CSBD9-DOX.

Finally, we compared imaging fluorescently labelled-C1-3 after administration of C1-3-Lipo-DOX or DOX containing liposomes coated with the control ScAb CSBD9 (CSBD9-Lipo-DOX). Mice were CCl_4_ injured and then at 24 h were given C1-3-Lipo-DOX to deplete HM or CSBD9-lipo-DOX non-targeting, control liposomes. Mice were then given fluorescently labelled-C1-3 at 72 h post CCl_4_ and then IVIS imaged 6 h later. Data in Fig. 6A–B show a clear reduction in IVIS signal in the C1-3-Lipo-DOX treated mice, where HM are selectively depleted compared to the CSBD9-Lipo-DOX group. This imaging approach can be used to monitor selective depletion of HM by C1-3-Lipo-DOX. However, it is important to consider that C1-3 is both targeting liposomes to HM to induce their apoptosis as well as monitor HM numbers; therefore we cannot exclude the possibility some of the reduction in signal in this experiment may be due to internalisation or blocking of synaptophysin by C1-3-Lipo-DOX. *Ex vivo* imaging of organs confirmed that the fluorescent signal is restricted to the liver and is higher in the CSBD9-Lipo-DOX group (Fig. 6C–D). HM depletion in mice receiving C1-3-Lipo-DOX was confirmed by a reduction in αSMA+ area (Fig. 6E–F). Serum transaminases were elevated equally between both groups confirming that the liver injury was similar (Supplementary Table 1).

## Discussion

Molecular optical imaging of fluorescent probes for *in vivo* studies permits non-invasive longitudinal assessment of biological processes and uses fewer animals compared to traditional preclinical models where multiple time-points are needed to evaluate disease progression or assess therapeutic effects. This technology provides insight into disease pathogenesis; however, there are limitations and technical hurdles that need to be overcome when developing a new fluorescence-based imaging method [30], [31]. In whole animals, *in vivo* fluorescence imaging is limited by tissue thickness, auto-fluorescence quenching and light scattering, making visualisation of deep-seated organs such as the liver challenging [32]. C57Bl/6 mice are often the preferred strain for preclinical mouse models because the majority of transgenic mice are generated on this background. However, fluorescent imaging of C57Bl/6 mice is difficult, black skin and fur absorbs light causing signal attenuation resulting in lower signal to noise ratio. Shaving mice can alleviate this problem but shaving disrupts the hair growth cycle causing increased melanin deposition and dark skin pigmentation [33]. Using different strains e.g*.*, white haired BALB/c mice or imaging C57Bl/6 mice with NIR probes, which reduce signal attenuation and scattering, can help overcome this problem.

We used an 800 nm protein labelling kit to conjugate a NIR fluorophore to C1-3 to monitor HM numbers in C57Bl/6 mice with liver fibrosis, highlighting the potential of this technology as a non-invasive imaging tool. NIR protein conjugation kits are commercially available; therefore *in vivo* fluorescence molecular imaging is potentially only limited by the identification of a target and availability of an antibody, ligand or peptide that binds the target. As such, this imaging strategy could be applied to other animal disease models. It is important to note that labelling efficiency of the fluorescent dye to the antibody can vary between labelling reactions. To minimise any potential variation in longitudinal studies and compare average radiant efficiency and HM numbers we have synthesised and labelled large quantities of antibodies in a single batch. Whilst absolute quantification of HM numbers in the injured liver by IVIS imaging is not achievable, this technology allows researchers to monitor stepwise changes in relative HM numbers within the same animal over the course of liver disease development. Furthermore, the changes in HM numbers can be directly compared between several experimental groups of animals within the same experiment.

We report for the first time that optical imaging of fluorescently labelled-C1-3 successfully monitors HM numbers in acute and chronic liver fibrosis models in C57Bl6 mice *in vivo.* IVIS imaging of acute injured WT and *cRel*^−/−^ mice that develop less fibrosis demonstrated that this molecular imaging approach identifies mice with fibro-protective phenotypes. Therefore, this imaging method could be used to screen and identify genetically modified mice, which are more or less susceptible to developing fibrosis without needing to confirm this histologically. Furthermore, we prove that this technology can monitor early response to therapies, which induce HM apoptosis or limit their activation/proliferation.

C1-3 mediated targeting of liposomes to HM could be exploited to deliver other payloads such as novel anti-fibrotic compounds that induce HM death or modulate their phenotype. Directly targeting drugs to HM to limit fibrosis *in vivo* without affecting other cell types is likely to improve drug efficacy and reduce side effects. Alternatively, C1-3-targeted-liposomes could be used to deliver siRNA or anti-sense oligonucleotides to selectively knockdown expression of fibrogenic regulators in HM in a temporal manner *e.g.* once fibrosis is established. This would help address the role of these genes/proteins in HM during fibrogenesis without the need to use costly and time-consuming genetic mouse models that rely on Cre deleter-mice to selectively remove the gene in HM.

*In vivo* fluorescent imaging is high throughput (5 animals/scan) and cost effective compared to other imaging modalities. We suggest that molecular imaging of HM using fluorescently labelled-C1-3 could be a useful tool for proof-of-concept studies or drug discovery studies where large numbers of compounds/doses are screened for a therapeutic effect or where an early predictor of therapeutic response is needed. *In vivo* imaging is now utilised as a translational vehicle between preclinical and early clinical studies. Recent advances in optical imaging cameras, narrower bandwidth filters and spectral un-mixing algorithms have improved IVIS sensitivity and the resolution is closer to positron emission tomography (PET) and single photon emission computed tomography (SPECT) [30], [31], [32]. In man liver biopsy is the gold standard procedure for the diagnosis and staging of liver fibrosis. However, liver biopsy has been criticised for its lack of reproducibility, interobserver and intraobserver variability and patient intolerability.

C1-3 binds to human HM, therefore C1-3 has the potential to be translated to man and used as an *in vivo* diagnostic agent when combined with clinically translatable imaging modalities such as PET or SPECT. Clinical imaging of C1-3 either alone or with existing serum fibrosis markers could be employed to help identify patients with progressive disease or predict an early effective therapy in clinical trials. Alternatively, C1-3 could be engineered to selectively deliver drugs to HM, which would reduce off-target adverse effects caused by a systematic administration of the drug, or improve compound efficacy.

## Financial support

This work was funded by the UK Medical Research Council (10.13039/501100000265MRC) (Grant MR/K0019494/1 to D.A.M and G0900535 to F.O.). A European Commission FP7 grant ‘INFLA-CARE’ (EC Contract No. 223151; http://inflacare.imbb.forth.gr/) to D.A.M, M.P. and others. D.A.M and F.O are funded by the cross-council Lifelong Health and Wellbeing initiative, which is managed by the MRC (award reference i L016354). HM was funded by the Hunter Memorial Fund. Partially supported by 10.13039/501100005010AIRC IG 2012 Grant IG12994 to P.P. and AIRC 2013 Grant IG14231 to M.P, D.D.P. partially supported by award from Fondazione Umberto Veronesi. The IVIS spectrum was purchased under a Wellcome Trust Equipment Grant (087961) awarded to D.A.M and others.

## Conflict of interest

M.C.W. has a financial interest in any commercial exploitation of the C1-3 antibody through any licensing agreement of a previous employer but takes no part and has no influence on any commercialisation activities. All other authors declared that they do not have anything to disclose regarding funding or conflict of interest with respect to this manuscript.

## Authors’ contributions

S.L. performed the majority of the laboratory-based work and analyses presented in the manuscript. S.J.H, J.L, H.L.M and H.B. performed a portion of the laboratory experiments and their related analyses. D.D.P, P.P, C.B and M.P generated and provided liposomes. M.C.W, M.P and D.A.M., provided advice and contributed to the experimental design. S.L and F.O. conceived the studies, designed the experiments and wrote the manuscript. All authors read and commented on the final manuscript.
