# Supplementary material for: A new fluorescence-based optical imaging method to non-invasively monitor hepatic myofibroblasts in vivo
Source: J Hepatol. 2016 Jul;65(1):75–83. doi: 10.1016/j.jhep.2016.03.021 (PMC4914605; doi:10.1016/j.jhep.2016.03.021)
Supplement: Supplementary data [file mmc1.pdf]

A new fluorescence based optical imaging method to non-invasively monitor hepatic myofibroblasts in vivo

Saimir Luli, Daniela Di Paolo, Patrizia Perri, Chiara Brignole, Stephen J. Hill, Helen Brown, Jack Leslie, Marshall HL, Matthew C Wright, Derek A Mann, Mirco Ponzoni, Fiona Oakley

Table of contents

Supplementary materials and methods……………………………………………………..2

Supplementary Fig. 1…………………..…………………………………………………..7

Supplementary Fig. 2…………………..…………………………………………………..9

Supplementary Fig. 3…………………..……………………………………………………10

Supplementary Fig. 4…………………..……………………………………………………12

Supplementary Fig. 5…………………..……………………………………………………14

Supplementary Fig. 6…………………..……………………………………………………15

Supplementary Fig. 7…………………..……………………………………………………16

Supplementary Fig. 8…………………..……………………………………………………18

Supplementary movie file legends……..…………………………………………………19

Supplementary Table. 1……………….……………………………………………………20

Supplementary Table. 2……………….……………………………………………………21

References…………………………….………………………………………………………22

Supplementary materials and methods

Liposome reagents and chemicals: Hydrogenated soy phosphatidylcholine (HSPC), cholesterol (CHE), 1,2-distearoyl-glycero-3-phosphoethanolamine-N-[methoxy(polyethylene glycol)-2000] (DSPE-PEG_{2000}), 1,2-distearoyl snglycero-3-phosphoethanolamine-N-[methoxy(polyethylene glycol)-2000] modified with a maleimido group at the distal terminus chain (1,2-distearoyl-sn-glycero-3-phosphoethanolamine-N-[maleimide(polyethylene glycol)-2000], DSPE-PEG_{2000}-MAL), were purchased from Avanti Polar Lipids (Alabaster, AL). Nuclepore polycarbonate membranes (pore sizes: 0.4, 0.2, and 0.1 µm) were purchased from Avestin (Ottawa, ON, Canada). 2-Iminothiolane (Traut's reagent) was obtained from Sigma (Sigma Chemical, St Louis, MO). Protein A/G column, ImmunoPure IgG elution buffer was purchased from Thermo Scientific-Pierce (Rockford, IL). Bio-Rad Protein Assay Reagent was purchased from Bio-Rad Laboratories (Mississauga, ON, Canada). Sephadex G-50 and Sepharose CL-4B, Doxorubicin hydrochloride (DOX) were purchased from Sigma.

Doxorubicin Stealth Liposomal preparation: Unilamellar stealth liposomes are synthesized from HSPC:CHOL:DSPE-PEG_{2000}, 2:1:0.01 molar ratio (liposomes without C1-3/CSDB9 targeting), HSPC:CHOL:DSPE-PEG_{2000}- DSPE-PEG_{2000}-MAL, 2:1:0.06:0.04 molar ratio (liposomes with C1-3/CSDB9 targeting). The lipids are first dissolved in chloroform and then combined in appropriate ratios. In some preparations, [^{3}H]CHE was added as a non-exchangeable, non-metabolizable lipid tracer. After evaporation under nitrogen, the thin lipid film is hydrated at 10mM phospholipids (PL) in about 1mL of 155 mM ammonium sulfate, pH 5.5 and then the hydrated liposomes are sequentially extruded through a series of polycarbonate filters of pore sizes ranging from 0.4 to 0.1 µm to produce primarily unilamellar vesicles. The liposomal mixture is eluted through a Sephadex G-50 column with sodium acetate buffer (100 mM $NaCH_3COO$, 140 mM NaCl) pH 5.5, DOX is then loaded into the liposomes via an ammonium sulfate gradient, using a method adapted from Bolotin [1] and

previously reported by Moase [2]. The mixture is then applied to a Sephadex G-50 column eluted with Hepes buffer (25 mM Hepes, 140 mM NaCl), pH7.4, to remove the unloaded DOX (DOX-liposomes) or to exchange the buffer (empty liposomes). The loading efficiency of DOX is around 95% and liposomes routinely contained DOX at a concentration of 150–180 µg DOX/µmol PL [3]. Finally, C1-3 or CSDB9 scFv fragments are coupled to the maleimide terminus of DSPE-PEG$_{2000}$-MAL using the previously described methods of coupling for whole antibodies and for Fab' fragments with slightly modifications [4, 5].

Briefly, to activate the C1-3 and CSDB9 fragments for reactivity toward the maleimide, we utilized 2-iminothiolane (Traut's reagent) to convert exposed amino groups on the antibody into free sulfhydryl groups. A 20:1 mole ratio of 2-iminothiolane to ScFv fragments and 1 hour of incubation at room temperature with occasional mixing gave optimal ScFv activation. After separation of thiolated ScFv from iminothiolane, with the use of Sephadex G-25 column chromatography, the ScFv was slowly added to the liposomes in the presence of a small magnetic stirring bar. Oxygen was displaced by running a slow stream of nitrogen over the reaction mixture. The tube was capped and sealed with Teflon tape, and the reaction mixture was incubated overnight at room temperature with continuous slow stirring. The resulting immunoliposomes were separated from unreacted ScFv by chromatography with the use of Sepharose CL-4B, sterilized by filtration through 0.2-µm pore cellulose membranes (Millipore Corp., Bedford, MA), and stored at 4 °C until use. The antibody density was evaluated by BioRad (Richmond, CA) protein assay.

Particle size (in nm), polydispersity index (PdI) and zeta potential (Z-potential in mV) of liposomal preparations were measured at 25 °C using a Malvern Nano ZS90 light scattering apparatus (Malvern Instruments Ltd., Worcestershire, UK), at a scattering angle of 90°C [6-10]. Zeta potential values were recorded following dilution in distilled water or in PBS. These results are presented as the average values ± standard deviation.

Unilamellar Liposomes possess a hydrodynamic diameter of about 155 nm (C1-3-lipo (DOX): 157.2 ± 3.164 nm; CSDB9: 158.2 ± 3.72 nm, with a mean PdI value 0.054 ± 0.025 for C1-3 and 0.072 ± 0.027 for CSDB9, indicating a very good monodisperse liposomal preparation. Moreover, the *Z*-potential values in water as well as in PBS were similar for the two formulations: C1-3 -25.8 ± 1.48 mV and 2.1 ± 0.665 mV and CSBD9 -29.2 ±2 .4 mV and -3 ± 0.53, respectively; indicating good stability properties in a medium resembling physiological conditions. These two parameters, taken together, warrant loss of aggregation during liposomes storage, and possibly, increased half-life in systemically conditions as expected due to the PEG shield [11].

Cellular binding studies *in vitro*: ^{3}H[CHE]-labeled liposomes were used to measure cellular binding, as described previously [3, 10, 12]. Briefly, cells (1×10^{6}/mL) were incubated with 600 nmol PL/mL of [3H]CHE-empty liposomal formulations, with or without coupled C1-3 and maintained at 4°C in a total volume of 200 µL. After a 1-hour incubation, the cells were extensively washed, and lysed with 1 N NaOH for evaluation of protein and for β-counting.

Analysis of *in vivo* fluorescent imaging: ROI analyses were limited to the upper quadrant of the abdomen, where the liver is anatomically (from the sternum to the middle of the abdominal region, with the abdominal region defined as the base of the sternum to the top of the pelvis). Anatomical localisation of the liver was confirmed by performing 3D Fluorescent Imaging Tomography (FLIT) reconstructions. Co-registration analysis of fluorescent signal from C1-3 (upper quadrant of abdomen) with a generic mouse organ atlas demonstrates the co-localisation of fluorescent signal with the mouse liver. To assess inter-observer variability when positioning ROIs, images were analysed blind and a correlation analysis was performed between two observers (suppl figure 8).

In vivo activated HSC isolation: Mice were given CCl_4 biweekly at (1:3 v/v CCl4:Olive Oil) for

two weeks, 24h after the final injection mice were humanely killed and then the livers removed. *In vivo* activated HM were then isolated as previously described by [13]

SDS-PAGE: Total protein was fractionated by 9% SDS-PAGE, transferred onto nitrocellulose and then blocked blots with Tris-buffered saline and Tween 20 (0.1%) containing 5% BSA before incubation overnight with primary antibodies to synaptophysin (Ab32127 Abcam, 1:2000 dilution), αSMA (A5228 Sigma, 1:2000 dilution) or GAPDH (Ab22555 Abcam, 1:1000 dilution). Next day membranes were washed in T-TBS and then incubated with horseradish peroxidase conjugated rabbit anti-mouse IgG (αSMA) mouse anti-rabbit IgG. (synaptophysin and GAPDH) were used at 1:2000 dilution. Blots were washed and antigen was detected using enhanced chemiluminescence (Amersham Biosciences).

Mouse Hepatic stellate cell isolation: Mouse hepatic stellate cells were isolated from C57Bl6 mice as previously described [14]

Sirius Red: Formalin-fixed and paraffin-embedded sections were stained with H&E, 0.1% Sirius Red Picric solution following standard procedures.

Immunohistochemistry: Staining was performed on formalin-fixed paraffin-embedded 5μm cut sections. Sections were dewaxed, dehydrated then endogenous peroxidase was blocked with 2% H2O2 in methanol. Antigen retrieval was achieved using proteinase K (20ug/ml) for detection of CD3 (MCA1477 serotec) at 1:100 dilution, F4/80 (ab6640 Abcam) at 1:100 dilution, 0.01% pronase for NIMP-R14 (ab2557 Abcam) at 1:200 dilution, trypsin for PCNA (ab2426 Abcam) at 1:250 dilution and Vector antigen unmasking for αSMA (F3777 Sigma) at 1:1000 dilution and CK-19 (ab84632 Abcam) at 1:250 dilution. Tissue sections were then blocked using an Avidin/Biotin Blocking Kit (Vector Laboratories) followed by 20% swine serum in PBS. Sections were then incubated with primary antibodies overnight at 4°C. The next day the slides were washed and incubated for 1 hour with a biotinylated secondary antibody: 1:200 dilution of goat anti-rat (STAR80B Serotec) for F4/80 and NIMPR14; swine anti-rabbit (E0353

Dako) for PCNA and CK-19 or goat anti-fluorescein (BA-0601, Vector Laboratories) for αSMA. Sections were washed and then incubated with R.T.U VECTASTAIN® kit for 1Hr and the antigen was visualised with 3, 3'-Diaminobenzidine (DAB). Prior to dehydration and mounting sections were counterstained with Mayer's Haematoxylin.

Immunocytochemistry: Cells were isolated from C75Bl6 murine livers and culture activated. Hepatic myofibroblasts were used for ICC staining between passages 1-4. Briefly, 22mmx22mm coverslips were sterilised with 70% ethanol and placed into a well of a 6 well plate. Cell numbers were determined using a haemocytometer and an equal number of cells seeded per well. Once ~70% confluency was reached, cells were incubated with 60μg/ml C1-3-AF for 2Hrs in HEPES/HBSS for 2Hrs at 37°C. Following this cells were fixed with 2% formaldehyde/0.2% glutaraldehyde in PBS and permeabilised with ice cold methanol. Cells were co-stained with aSMA-FITC. Finally, cell nuclei were stained with DAPI, mounted on slides and visualised using a Zeiss fluorescent microscope. Images taken at x200 magnification.

Image Analysis: Slides were imaged using a Nikon ECLIPSE Ni-U (Nikon Corporation) microscope. At least 12 fields were manually counted for NIMP-R14 and PCNA staining at ×20 magnification. Sirius Red, α-SMA, and F4/80 staining were analysed using Nikon Imaging Software Elements Basic Research (NIS-Elements Br, Nikon) a minimum of 12 fields (x20 magnification) were captured unless otherwise stated.

RNA Isolation and Real-Time Polymerase Chain Reaction: Total RNA was isolated from mouse livers using the Total RNA Purification Kit (QIAGEN). RNA was quantified using the NanoDrop. Complementary DNA was synthesized using a Promega kit (Promega). Real-time polymerase chain reaction was performed with SYBR Green as per manufacturer's instructions using the primers listed in supplemental table 2.

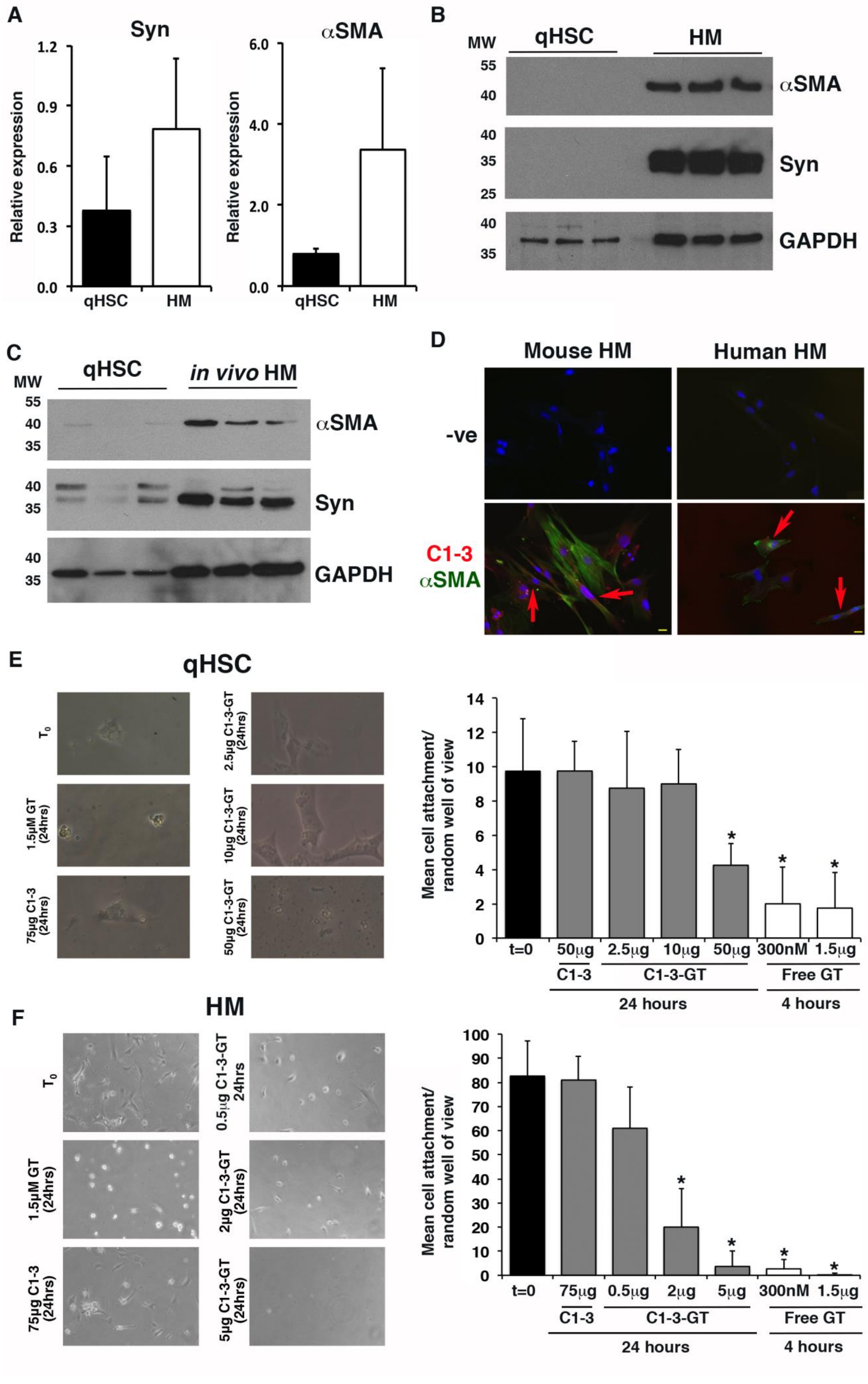

A
Syn
Relative expression
1.2
0.9
0.6
0.3
0.0
qHSC
HM
αSMA
6.0
4.0
2.0
0.0
B
MW
qHSC
HM
55
40
αSMA
35
25
Syn
GAPDH
C
in vivo HM
D
Mouse HM
Human HM
-ve
C1-3
αSMA
E
qHSC
T0
1.5µM GT (24hrs)
75µg C1-3 (24hrs)
2.5µg C1-3-GT (24hrs)
10µg C1-3-GT (24hrs)
50µg C1-3-GT (24hrs)
Mean cell attachment/ random well of view
14
12
10
8
6
4
2
0
t=0
50µg
2.5µg
10µg
50µg
300nM
1.5µg
C1-3
C1-3-GT
Free GT
24 hours
4 hours
F
HM
0.5µg C1-3-GT 24hrs
2µg C1-3-GT (24hrs)
5µg C1-3-GT (24hrs)
100
90
80
70
60
50
40
30
20
10
75µg
0.5µg
2µg
5µg

Supplementary Fig. 1. Synaptophysin is expressed in activated HM and C1-3-GT is relatively ineffective at killing qHSC *in vitro* in contrast to culture-activated HM. (a) Relative mRNA expression of synaptophysin (Syn) and αSMA in qHSC and *in vitro* activated HM. (b) Western blot analysis of synaptophysin (Syn), αSMA and GAPDH in qHSC and HM. (c) Western blot analysis of synaptophysin (Syn), αSMA and GAPDH in qHSC and *in vivo* activated HM. (d) Immunofluorescence images of mouse and human *in vitro* activated HM incubated with FITC conjugated C1-3 (green), fixed and then stained αSMA (Red). Red arrows denote col-localisation of C1-3 binding to HM and αSMA. (e) Murine HSC 24 hours after isolation (qHSCs), cells were washed and cultured in serum-free medium and treated as indicated. Representative images show cell morphology and attachment of t=0 cells ± treatment. Graph shows mean cell attachment in t=0 cells ± treatment. (f) Day 10 murine activated HSCs (HM) were washed and then cultured in serum-free medium and treated as indicated. Representative images show cell morphology and attachment in t=0 ± treatment. Graph shows mean cell attachment in t=0 cells ± treatment. Data are n=3 separate experiment using cells isolated from separate batches of mice. [*]**P* < 0.05 using an ANOVA.

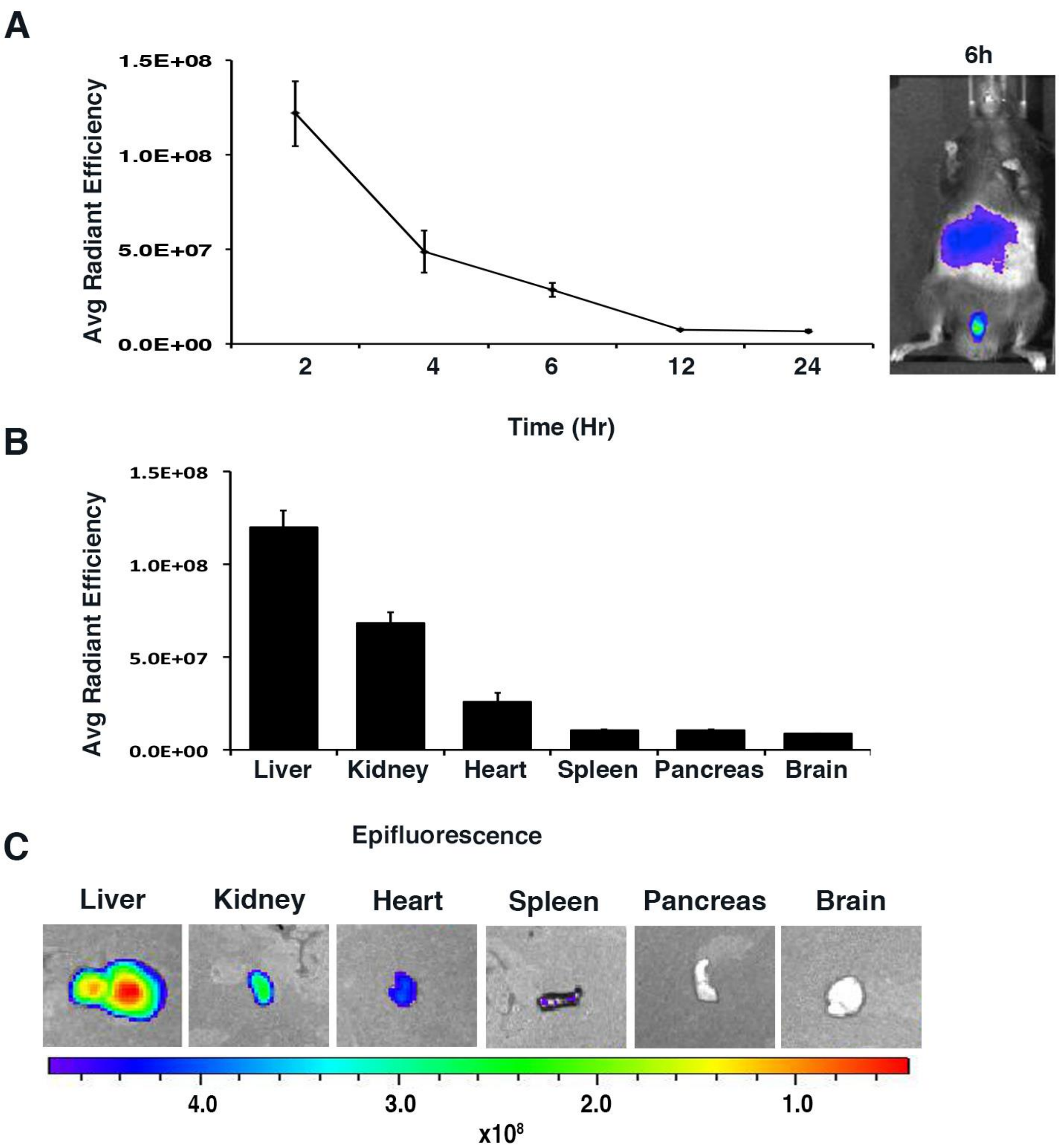

Supplementary Fig. 2. Biodistribution of fluorescently labelled-C1-3. (a) Graph showing average radiant efficiency calculated from whole body imaging of acute CCl_4 injured mice given fluorescently labelled-C1-3 over time and a representative picture of IVIS imaging of a mouse at 6Hr. (b-c) Representative *ex vivo* IVIS images and graph showing the average radiant efficiency in the brain, pancreas, spleen, heart, kidney and liver of uninjured WT mice. Data are expressed as means ± s.e.m, minimum of n=3/group.

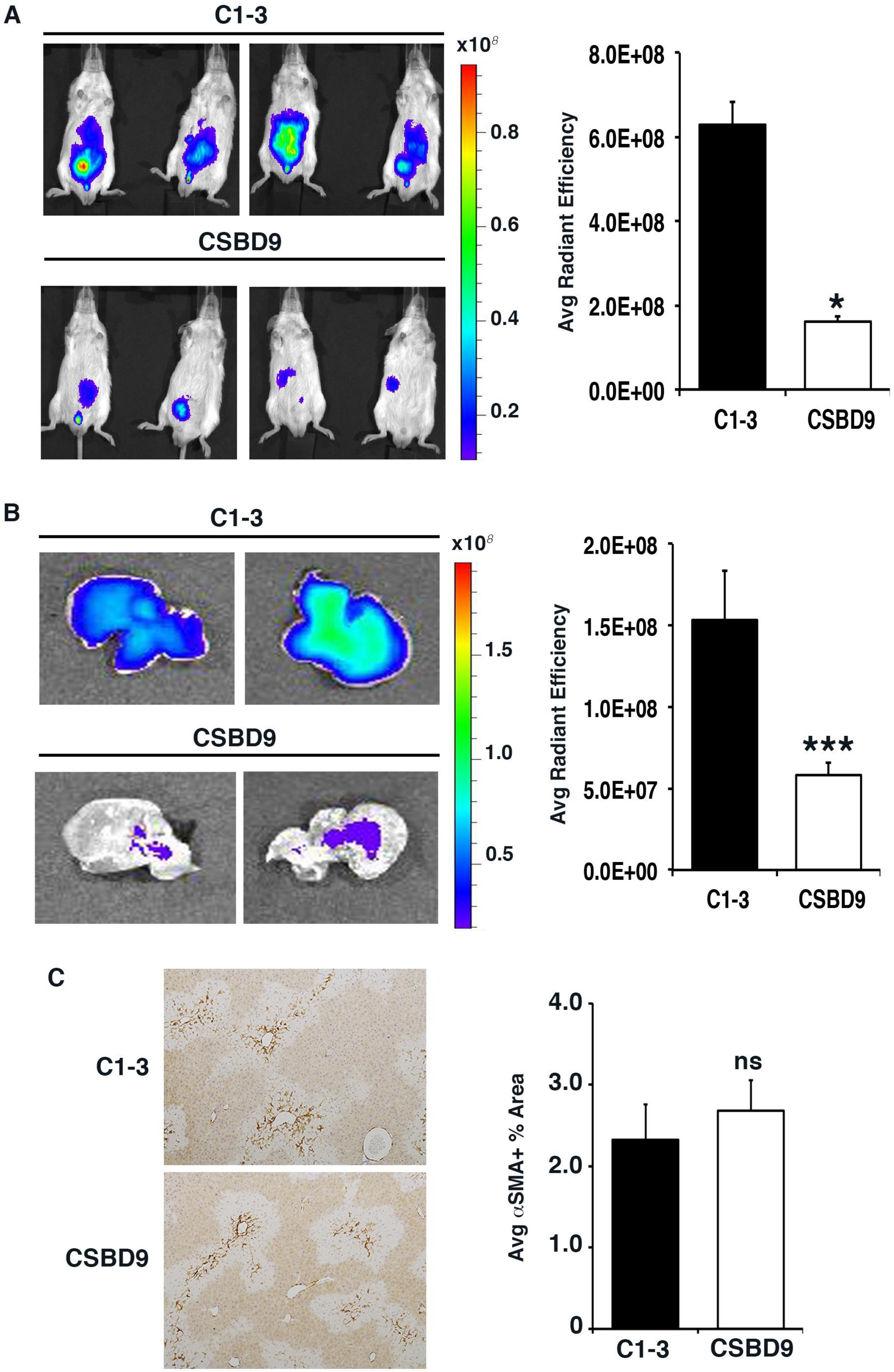
A
C1-3
CSBD9
x10^8
0.8
0.6
0.4
0.2
Avg Radiant Efficiency
8.0E+08
6.0E+08
4.0E+08
2.0E+08
0.0E+00
*
C1-3
CSBD9
B
C1-3
CSBD9
x10^8
1.5
1.0
0.5
Avg Radiant Efficiency
2.0E+08
1.5E+08
1.0E+08
5.0E+07
0.0E+00
***
C1-3
CSBD9
C
C1-3
CSBD9
Avg αSMA+ % Area
4.0
3.0
2.0
1.0
0
ns
C1-3
CSBD9

Supplementary Fig. 3. 3D reconstruction of fluorescently labelled-C1-3 or CSBD9 control antibody. (a) Representative picture of IVIS images and a graph showing average radiant efficiency calculated from whole body imaging of 48h CCl_4 injured BalBc mice given fluorescently labelled-C1-3 or CSBD9. (b) Representative *ex vivo* IVIS images and graph showing the average radiant efficiency from livers of 48h CCl_4 injured mice. (c) Photomicrographs (x100 magnification, 3x3 fields) and graph showing average αSMA+ area in liver sections from 48h CCl_4 injured WT mice, scale bars = 200 microns. Data are expressed as means ± s.e.m, n=4 mice/group. Unpaired t-test, *$P < 0.05$ and ***$P < 0.001$ compared to CSBD9 control.

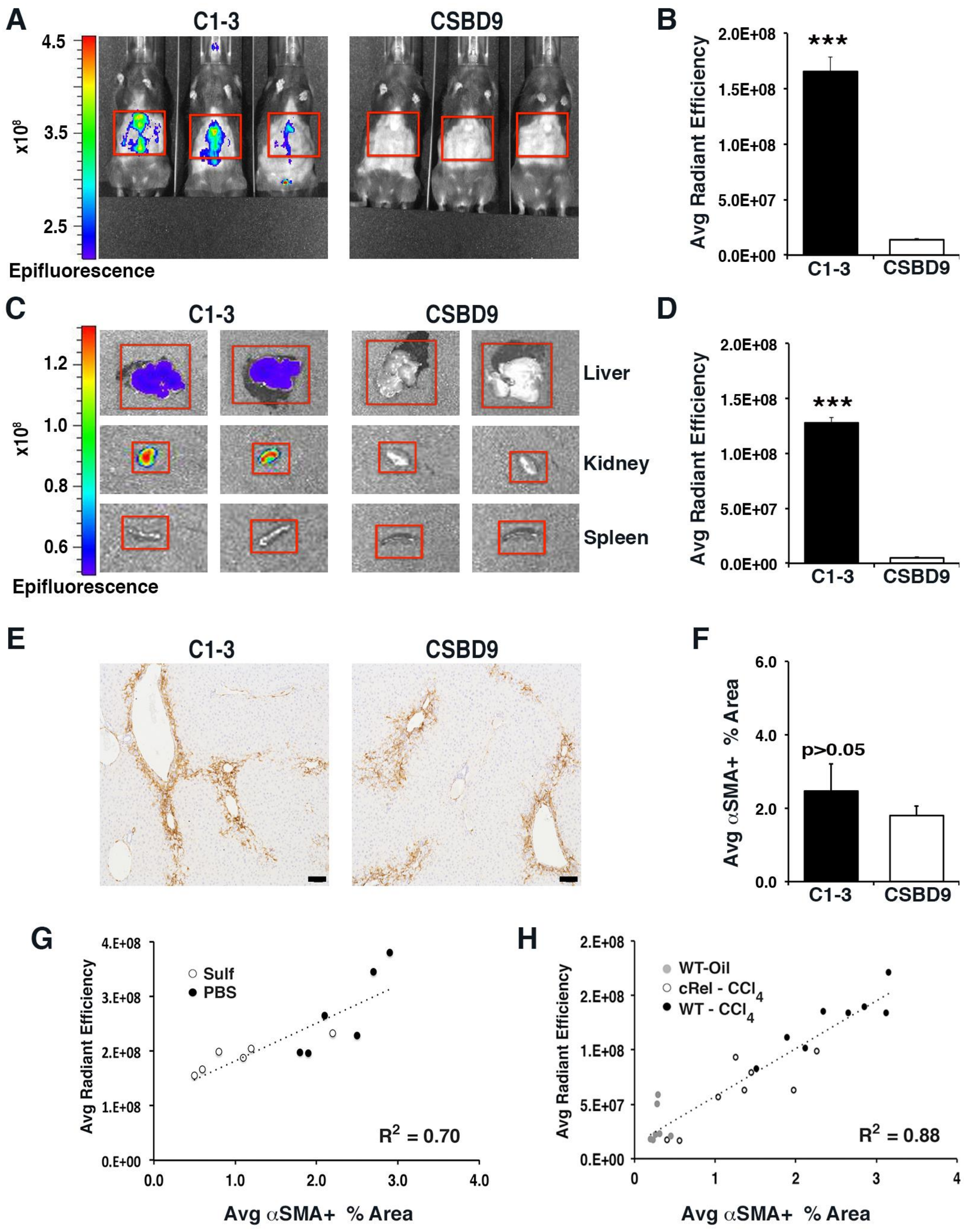

Supplementary Fig. 4. Fluorescently labelled-C1-3 but not -CSBD9 can be used to optically image hepatic myofibroblasts *in vivo* during acute liver injury. (a) IVIS images and (b) graph showing the average radiant efficiency of 48Hr acute CCl_4 injured WT mice

given fluorescently labelled C1-3 or CSBD9. (c) Representative *ex vivo* images of the liver (top), kidney (middle) and spleen (bottom) and (d) graph showing average radiant efficiency of *ex vivo* imaged acute CCl_4 livers given labelled C1-3 or CSBD9. (e) Photomicrographs (x100 magnification, 3x3 fields) and (f) graph showing average αSMA+ area in liver sections from acute CCl_4 injured WT mice, scale bars = 200 microns. Data are means $\pm$ s.e.m, minimum of n=3/group. Unpaired t-test, ***$P < 0.001$ compared to CSBD9 control. (g) Graphs show correlation analysis of αSMA+ stained area in livers from 48Hr acute CCl_4 injured WT mice treated +/- sulfasalazine plotted against average radiant efficiency and fitted with a linear regression, n=6 mice/group. (h) Graphs show correlation analysis of αSMA+ stained area in livers from 48Hr olive oil or acute CCl_4 injured WT or c-Rel knockout mice plotted against average radiant efficiency and fitted with a linear regression n= a minimum of 6 mice/group.

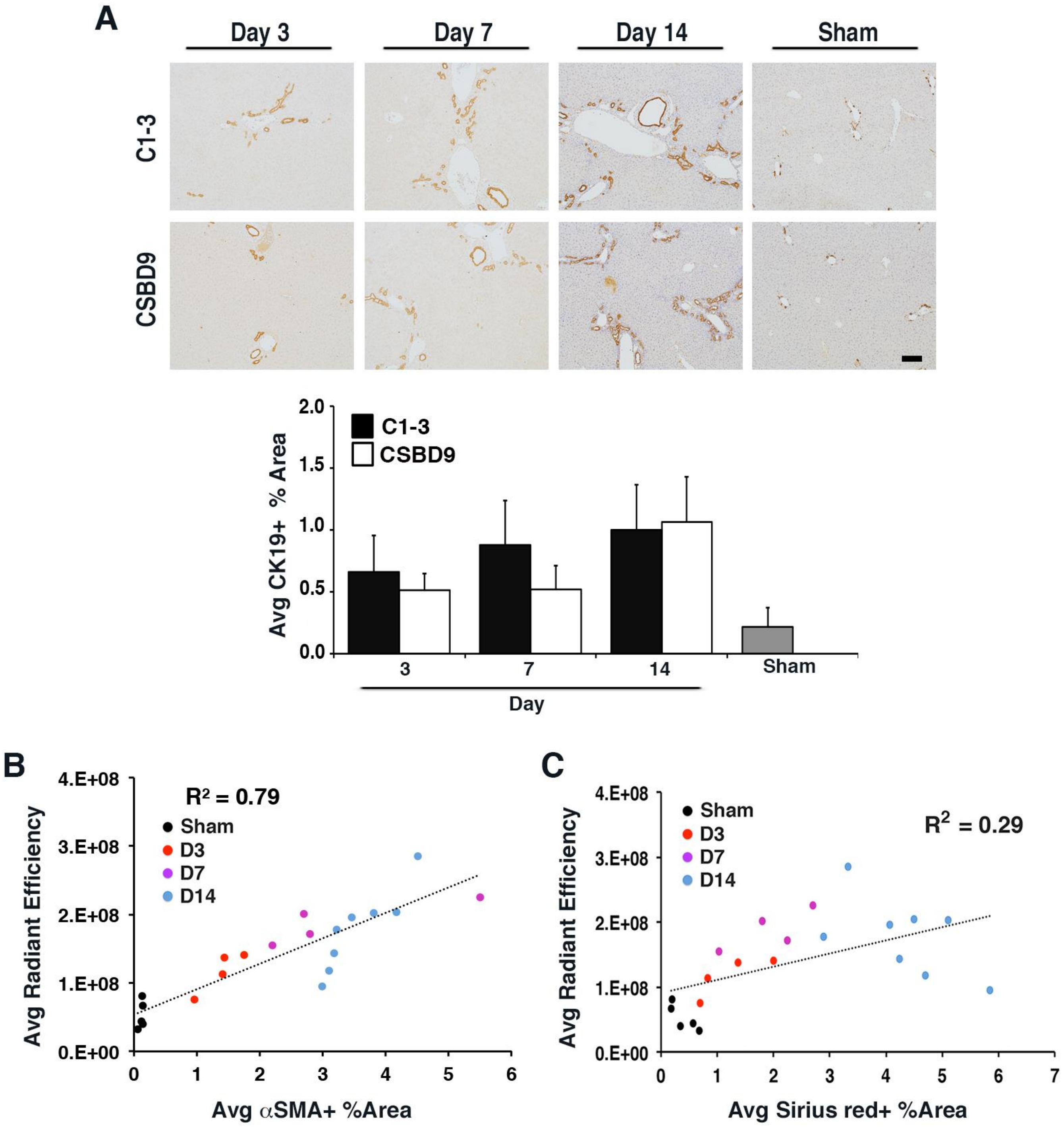

Supplementary Fig. 5. C1-3 imaging and histological analysis of BDL mice. (a) Representative photomicrographs at x100 magnification of CK19 staining and graph showing average CK19+ stained area in BDL or sham operated mice, (scale bar = 200 microns). (b-c) Graphs show correlation analysis of αSMA+ (b) or Sirius red+ (c) stained area in sham and day 3, 7 and 14 BDL livers plotted against average radiant efficiency and fitted with a linear regression. Data are means ± s.e.m, minimum of n=4-8/group.

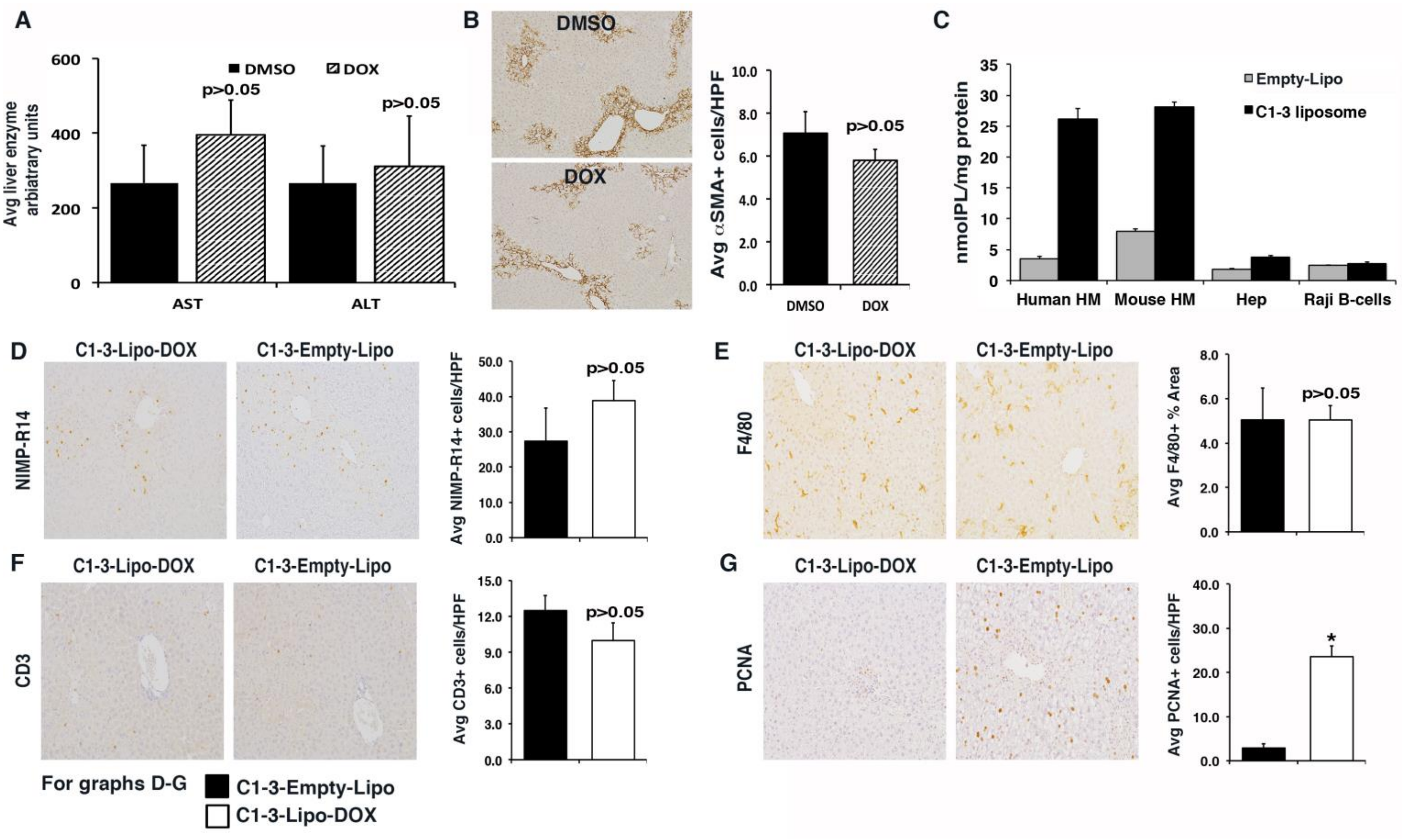

Supplementary Fig. 6. Liposomal depletion of HM does not alter hepatic inflammation but stimulates liver growth in acute liver injury. (a) Graph of average serum transaminases ALT and AST and (b) photomicrographs and graph show average αSMA+ stained area in liver sections from acute CCl_4 injured mice ± free doxorubicin (DOX). (c) Graph shows average binding of control-liposomes (uncoated, empty) or C1-3-liposomes to human and mouse HM, primary hepatocytes (Hep) and Raji B-cells using an in vitro binding assay. (d) Images (x200 magnification) of NIMP-R14+ neutrophils and graph showing average number of NIMP-R14+ cells/HPF in control or DOX loaded C1-3-liposomes. (e) Photomicrographs (x100 magnification) of F4/80+ macrophages and graph showing average F4/80+ stained area. (f) Images (x200 magnification) of CD3+ T-cells and graph showing average CD3+ cells/HPF. (g) Photomicrographs (x100 magnification) of PCNA+ cells and graph showing average PCNA+ hepatocytes/HPF. Data are means ± s.e.m, n=5/group. Unpaired t-test, **P* < 0.05 compared to control SLL.

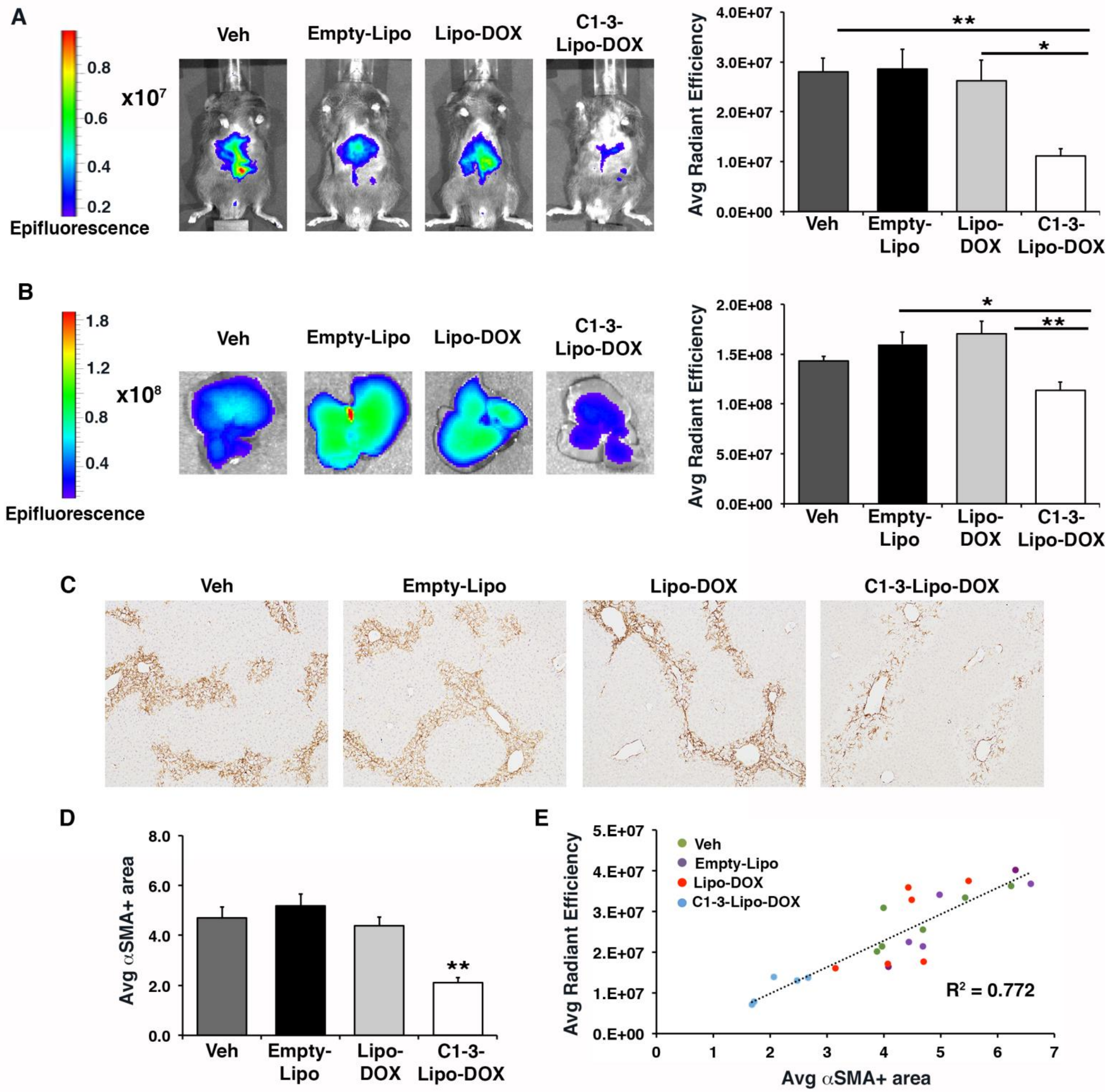

Supplementary Fig. 7. C1-3 IVIS imaging of mice with acute liver injury after liposome administration. (a-b) Representative IVIS and *ex vivo* images and graphs showing the average radiant efficiency of 72Hr acute CCl_4 injured WT mice treated with vehicle (CCl_4), empty liposomes (Empty-Lipo), liposomes containing DOX (Lipo-DOX) or C1-3 coated liposomes containing DOX (C1-3-Lipo-DOX) and imaged with fluorescently labelled C1-3. (c) Photomicrographs (x100 magnification, 3x3 fields) and (d) graph showing average αSMA+ area in liver sections from acute

CCl_4 injured WT mice after administration of liposomes (C1-3-Lipo-Dox is ** compared to all groups). (e) Graphs show correlation analysis of αSMA+ with IVIS imaging in acute CCl_4 injured WT mice after administration of liposomes. Data are means ± s.e.m, n=5-6/group for *in vivo* studies. ANOVA, *$P < 0.05$ and **$P < 0.01$ as shown.

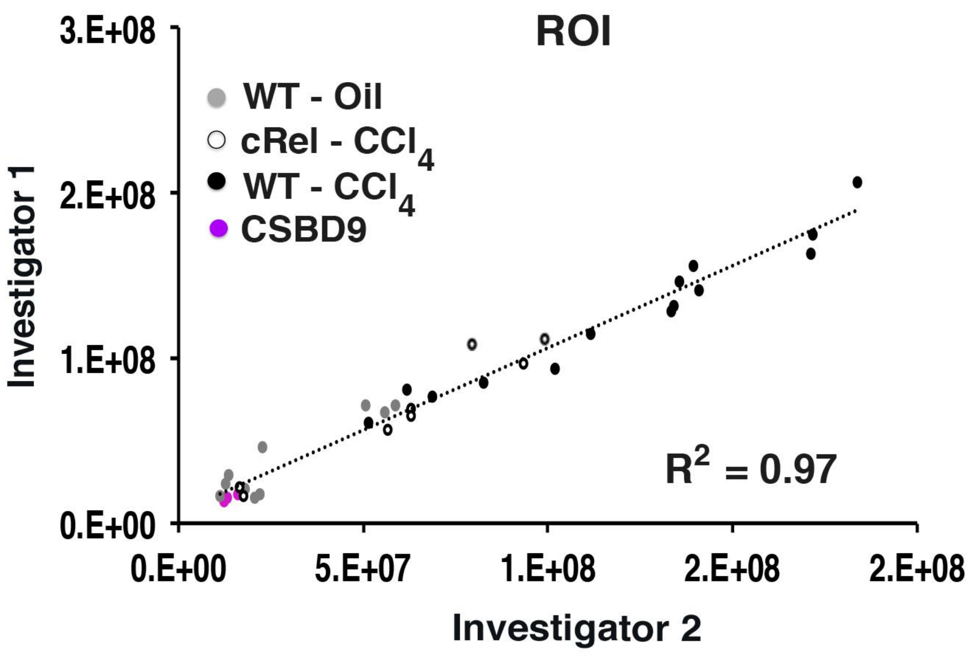

Supplementary Fig. 8. Inter-observer comparison. Graph shows a correlation of IVIS analysis expressed as average radiant efficiency generated from ROI placed by investigator 1 and investigator 2 in samples from acute CCl_4 or olive oil WT mice and acute CCl_4 c-Rel$^{-/-}$ mice given fluorescent labelled C1-3 and acute CCl_4 WT mice given CSBD9, each data point represents an individual animal.

Supplementary movie files

Movie file 1. Shows 3D reconstruction of Fluorescent Imaging Tomography (FLIT) scans of fluorescently labelled-C1-3 localisation in a 48Hr CCl_4 injured mouse. FLIT images are co-registered with the mouse organ atlas.

Movie file 2. Shows 3D reconstruction of Fluorescent Imaging Tomography (FLIT) scans of fluorescently labelled-CSBD9 localisation in a 48Hr CCl_4 injured mouse. FLIT images are co-registered with the IVIS mouse organ atlas.

Liver Function Test			
Study	ALP	ALT	AST
SHAM-C1-3	37.6 ± 8.3	17.2 ± 2.9	85.1 ± 12.7
SHAM-CSBD9	18.6 ± 2.9	21.0 ± 2.9	102.3 ± 31.4
3Day-BDL-C1-3	49.5 ± 12.5	106.5 ± 27.4	202.7 ± 40.7
3Day-BDL-CSBD9	44.0 ± 15.9	57.0 ± 15.3	187.5 ± 16.2
7Day-BDL-C1-3	178.0 ± 12.2	383.5 ± 79.9	684.0 ± 147.0
7Day-BDL-CSBD9	144.5 ± 21.8	72.5 ± 48.9	551.3 ± 74.4
14Day-BDL-C1-3	287.2 ± 25.3	159.2 ± 26.3	340.3 ± 49.2
14Day-BDL-CSBD9	358.7 ± 106.0	133.8 ± 27.1	279.9 ± 42.1
Acute CCl_4-C1-3		3084.7 ± 1545.2	2223.0 ± 863.2
Acute Oil-C1-3		136.7 ± 35.8	477.0 ± 67.1
Acute CCl_4-Sulf-C1-3		1799.1 ± 215.9	1223.4 ± 167.5
Acute CCl_4-PBS-C1-3		1795.6 ± 368.2	1684.3 ± 249.8
WT-Acute-CCL_4-C1-3		1216.6 ± 183.8	1016.2 ± 151.5
c-Rel$^{-/-}$-Acute-CCL_4-C1-3		730.0 ± 147.5	678.3 ± 110.4
WT-Acute-Oil-C1-3		30.9 ± 6.9	77.4 ± 9.5
Lipo-DOX-CCl4-C1-3-IVIS	83.4 ± 6.3	694.3 ± 161.9	1700.0 ± 197.1
Lipo-DOX-CCl4-CSBD9-IVIS	86.9 ± 5.2	404.0 ± 54.7	1518.3 ± 259.9
48Hr-Lipo-DOX-C1-3	129.0 ± 8.4	8284.8 ± 1106.9	5754.0 ± 835.3
48Hr-Lipo-Empty-C1-3	140.0 ± 5.3	4272.0 ± 2534.6	2889.0 ± 1112.6
72Hr-Lipo-DOX-C1-3	78.6 ± 8.2	327.6 ± 33.4	978.0 ± 186.6
72Hr-Lipo-Empty-C1-3	90.6 ± 15.7	1188.6 ± 503.9	1511.4 ± 383.0
5day-Lipo-DOX-C1-3	63.0 ± 6.4	126.8 ± 35.7	1672.5 ± 696.1
5day-Empty-DOX-C1-3	69.6 ± 5.6	111.6 ± 26.8	466.2 ± 53.5
Acute CCl_4		682.8 ± 184.5	681.6 ± 51.6
Acute CCl_4 Lipo-Empty		508.0 ± 67.6	1073.0 ± 197.1
Acute CCl_4 Lipo-DOX		881.5 ± 67.1	1176.5 ± 75.5
Acute CCl_4 Lipo-DOX-C1-3		828.0 ± 275.5	1551.6 ± 114.4

Supplementary Table 1: Liver function tests. Serum levels of alanine transaminase (ALT) and aspartate aminotransferase (AST) and alkaline phosphatase (ALP) were measured. Enzymes are measured in arbitrary units and data are means ± s.e.m.

Gene	Forward Primer 5’-3’	Reverse Primer 5’-3’	Annealing temperature °C
**α-SMA**	TCAGCGCCTCCAGTTCCT	AAAAAAAACCACGAGTAACAAATCAA	58
**TIMP-1**	GCAACTCGGACCTGGTCATAA	CGGCCCGTGATGAGAAACT	58
**TGF-β1**	CTCCCGTGGCTTCTAGTGC	GCCTTAGTTTGGACAGGATCTG	58
**Collagen 1**	TTCACCTACAGCACGCTTGTG	GATGACTGTCTTGCCCCAAGTT	58
**TNF α**	AGATGCTGGGACAGTGACCT	GATTTAGTGTGCTGAAAGCTCTACTC	58
**IL-1 β**	CAACCAACAAGTGTATATTCTCCATG	GATCCACACTCTCCAGCTGCA	58
**KC**	CTGGGATTCACCTCAAGAACATC	CAGGGTCAAGGCAAGCCTC	58
**MCP1**	AGGTCCCTGTCATGCTTCTG	TCTGGACCCATTCCTTCTTG	58
**GAPDH**	GCACAGTCAAGGCCGAGAAT	GCCTTCTCCATGGTGGTGAA	58
**Synaptophysin**	ATGGACGTGGTGAATCAGCTGGT	GGGTGCATCAAAGTACACTTGG	63
**18S**	CCCGAAGCGTTTACTTTGAA	CCCTCTTAATCATGGCCTCA	60

Supplementary Table 2: Table of primer sequences and annealing temperatures used for mouse quantitative RT-PCR.

References:

[1] Bolotin EM, Cohen R, Bar LK, Emanuel N, Ninio S, Barenholz Y, et al. Ammonium sulfate gradients for efficient and stable remote loading of amphipathic weak bases into liposomes and ligandoliposomes. Journal of Liposome Research 1994;4:455-479.

[2] Moase EH, Qi W, Ishida T, Gabos Z, Longenecker BM, Zimmermann GL, et al. Anti-MUC-1 immunoliposomal doxorubicin in the treatment of murine models of metastatic breast cancer. Biochimica et Biophysica Acta - Biomembranes 2001;1510:43-55.

[3] Pastorino F, Brignole C, Marimpietri D, Sapra P, Moase EH, Allen TM, et al. Doxorubicin-loaded Fab' fragments of anti-disialoganglioside immunoliposomes selectively inhibit the growth and dissemination of human neuroblastoma in nude mice. Cancer Res 2003;63:86-92.

[4] Pastorino F, Stuart D, Ponzoni M, Allen TM. Targeted delivery of antisense oligonucleotides in cancer. J Control Release 2001;74:69-75.

[5] Pagnan G, Montaldo PG, Pastorino F, Raffaghello L, Kirchmeier M, Allen TM, et al. GD2-mediated melanoma cell targeting and cytotoxicity of liposome-entrapped fenretinide. Int J Cancer 1999;81:268-274.

[6] Loi M, Marchio S, Becherini P, Di Paolo D, Soster M, Curnis F, et al. Combined targeting of perivascular and endothelial tumor cells enhances anti-tumor efficacy of liposomal chemotherapy in neuroblastoma. J Control Release 2010;145:66-73.

[7] Pastorino F, Brignole C, Marimpietri D, Cilli M, Gambini C, Ribatti D, et al. Vascular damage and anti-angiogenic effects of tumor vessel-targeted liposomal chemotherapy. Cancer Res 2003;63:7400-7409.

[8] Di Paolo D, Pastorino F, Zuccari G, Caffa I, Loi M, Marimpietri D, et al. Enhanced anti-tumor and anti-angiogenic efficacy of a novel liposomal fenretinide on human neuroblastoma. J Control Release 2013;170:445-451.

[9] Di Paolo D, Ambrogio C, Pastorino F, Brignole C, Martinengo C, Carosio R, et al. Selective therapeutic targeting of the anaplastic lymphoma kinase with liposomal siRNA induces apoptosis and inhibits angiogenesis in neuroblastoma. Mol Ther 2011;19:2201-2212.

[10] Di Paolo D, Brignole C, Pastorino F, Carosio R, Zorzoli A, Rossi M, et al. Neuroblastoma-targeted nanoparticles entrapping siRNA specifically knockdown ALK. Mol Ther 2011;19:1131-1140.

[11] Li SD, Huang L. Stealth nanoparticles: high density but sheddable PEG is a key for tumor targeting. J Control Release 2010;145:178-181.

[12] Pagnan G, Stuart DD, Pastorino F, Raffaghello L, Montaldo PG, Allen TM, et al. Delivery of c-myb antisense oligodeoxynucleotides to human neuroblastoma cells via disialoganglioside GD(2)-targeted immunoliposomes: antitumor effects. J Natl Cancer Inst 2000;92:253-261.

[13] De Minicis S, Seki E, Uchinami H, Kluwe J, Zhang Y, Brenner DA, et al. Gene expression profiles during hepatic stellate cell activation in culture and in vivo. Gastroenterology 2007;132:1937-1946.

[14] Oakley F, Mann J, Nailard S, Smart DE, Mungalsingh N, Constandinou C, et al. Nuclear factor-kappaB1 (p50) limits the inflammatory and fibrogenic responses to chronic injury. The American journal of pathology 2005;166:695-708.
